# Heterogeneous knowledge spillover channels in universities and green technology innovation in local firms: Stimulating quantity or quality?

**DOI:** 10.3389/fpsyg.2022.943655

**Published:** 2022-10-10

**Authors:** Libing Nie, Hong Gong, Danxiao Zhao, Xiuping Lai, Mengyue Chang

**Affiliations:** ^1^Economics and Management School, Wuhan University, Wuhan, China; ^2^Research Center for China Industry-University-Research Institute Collaboration, Wuhan University, Wuhan, China; ^3^Research Center of Strategic Emerging Industries, Wuhan University, Wuhan, China; ^4^School of Business, Nanjing University, Nanjing, China

**Keywords:** heterogeneous knowledge spillover channel, university patents, subsequent innovation, innovation quantity, innovation quality

## Abstract

Sluggish status of green technology development has stimulated research into new incentives and pathways. Beyond the traditional regulatory-push and demand-pull approaches, we reposition the strength of the technology push. Based on the innovation diffusion theory, a multidimensional path model of knowledge spillover in universities is constructed, and the impact of heterogeneous knowledge spillover channels on green innovation activities of local firms is discussed. We find that R&D collaboration has a significant effect on local firms' quality but not the quantity of green innovation. Contrarily, patent citations and technology transfer have unequal positive effects on the quantity of green innovation of local firms, while there is no evidence that they can also improve the quality of green innovation. Despite regional disparities, strict environmental regulations are pushing companies to cite university patents in some regions. The university knowledge stock has largely contributed to both quantitative and qualitative advances in subsequent green innovation in local firms. Our conclusions provide a precise and objective evaluation of the impact mechanism of multiple knowledge spillover channels in universities on firms' green innovation, as well as a reference for the selection of the form of industry–university–research collaboration.

## Introduction

Although green technologies are acknowledged as promising in addressing environmental pollution and building a harmonious society, they are still lagging behind the interest in them. Over the years, the proportion of green patents of total patents in China has averaged only 2–3% (Wang and Zhao, 2019). Environmental regulation and government subsidies have long been viewed as significant apparatuses for encouraging green technologies, and have been the focus of scholastic consideration. Yet, the role of these instruments is limited, as evidenced by the increase of green patents (Horbach et al., 2012). Additional powerful tools are urgently needed to advance green innovations (Koch and Simmler, 2020).

As the primary location of scientific research and technological innovation, the accumulation of resources in universities has always been a self-evidently supportive force for the growth of technological innovation in society (Qiu et al., 2017). Does the positive impact of university R&D on firm innovation, however, applies equally to green innovation? Given the complexity and profitability uncertainty of green innovation (Barbieri et al., 2020), there are valid reasons to be justified in wondering whether this is true. Specifically, on the one hand, green innovation is characterized by high complexity, high cost, high risks, and long cycles, which is difficult for firms to complete alone. Firms tend to cooperate closely with universities, research institutes, and other innovation subjects. On the other hand, green innovation is different from the traditional technology trajectory with a strong frontier and novelty. It is hard for firms to absorb the green innovation achievements of universities timely and fully. The absence of reflection on this question is incredible and is not conducive to the sustained depth and spread of green innovation. Hence, the first question we are going to address is to reveal whether the green innovation activities of universities have aided green innovation in firms.

Prevailing research on the promotion of green technology innovation has largely concentrated on the regulatory-push and demand-pull approaches (Horbach et al., 2012), whereas the power of technology push has been overlooked (Koch and Simmler, 2020). Among them, the knowledge spillover of universities, especially nearby universities, is an ongoing topic in the field of innovation. When it comes to this, scholars put more emphasis on industry–university–research synergy in a limited sense, investigating outcomes of collaboration like incentives, crowding-out effects, dispute resolutions, and benefit distributions for firms' innovation (Arza, 2010; Zhang et al., 2019; Vega-Jurado et al., 2021; Verre et al., 2021). In the wider sense, nevertheless, there are numerous forms of university-industry cooperation, such as the collaborative innovation center for explicit information flow and the knowledge reserve of universities for implicit knowledge flow. Scholars may have overlooked the heterogeneous impact of multiple spillover channels on local firms' innovations, such as patent citations, R&D collaboration, technology transfer, and knowledge stock.

There is also a quantitative and qualitative distinction in the measurement of green innovation. The quantity of innovation indicates the efficiency of the impact of knowledge spillovers, while the quality of innovation reflects the effect of the impact (Chen and Zhang, 2020). It is logical to distinguish the impacts of knowledge spillovers on the two. Existing studies either separate the two and focus on one or the other, or ignore the distinction between the two and adopt a unified measurement approach, with few studies concentrating on both of them (Hagedoorn and Cloodt, 2003; Lanjouw and Schankeman, 2004; Cai and Li-Ping, 2017).

Our research extends beyond determining whether the effects of university-based innovation activities on green innovation in local firms are still valid. Further, we classify the multiple channels of knowledge spillover from universities into R&D collaboration, patent citations, technology transfer, and knowledge stock. We seek to figure out the diverse effects of the different channels on the quantity and quality of green innovation in local enterprises, respectively. On the one hand, this might help clarify the role of green research and development in universities in terms of subsequent green innovation. On the other hand, it responds to the question of which type of collaboration local firms should pursue when confronted with the dual narrative of increased innovation quantity and quality. As a result, a more precise and efficient collaboration mechanism for green innovation can be developed, as well as more powerful growth paths.

Considering the challenges that universities may face in the process of green R&D output successfully promoting the development of green technology in society, this paper puts forward three research questions. Firstly, we seek to answer the problem that whether green innovation activities of universities can promote green technology development in the region; secondly, we try to identify the incentive effects of different knowledge spillover channels on the green development of society. We refine the heterogeneous knowledge spillover channels from universities, and divide them into patent citations, R&D collaboration, technology transfer, and knowledge stock; finally, from the perspective of efficiency and effectiveness, we identify the impact of different knowledge spillover channels on the quantity and quality of subsequent innovation. By answering these questions, we try to achieve the following objectives: on the one hand, our objective is to clarify the mechanism of green innovation activities in universities on subsequent innovation; on the other hand, we aim to answer the question of how firms can choose efficient university-industry cooperation when facing the dual demands of innovation quantity and quality improvement. In this way, it is possible to establish a more precise and efficient model of industry–university–research cooperation and a growth path for green innovation.

## Literature review

### Innovation activities in universities and enterprises

Innovation activities have obvious external effects, with knowledge spillover serving as an intermediary mechanism. The main generation carriers of this effect are universities and other public research institutions, and the receiving carriers include firms, individuals, etc. Knowledge spillover in university refers to the flow of knowledge and technical elements from universities to society (Ardito et al., 2019). The university technology transfer is an intermediate link between university basic research and firm application research. An important embodiment of its radiating social function as a science and technology source (Fukugawa, 2013; Messeni Petruzzelli and Murgia, 2020). As a significant contributor to industrial innovation in different fields of contemporary society (Mansfield and Lee, 1996), university R&D activities and subsequent knowledge spillover are conducive to promoting industrial innovation and regional economic growth (Barletta et al., 2017; Min et al., 2020). At the same time, firms cooperate with innovation subjects in different types and levels to better carry out innovation activities (Bell, 2005), because firm innovation performance is not only affected by its innovation ability, but also by external forces.

The impact of universities' R&D activities on industrial innovation capacity is reflected in two ways. On the one hand, universities participate in innovation activities as R&D subjects. They promote knowledge transmission and diffusion among innovation agents through industry–university–research collaboration and knowledge flow (Anselin et al., 2000), to optimize the combination of science and technology with other production factors and promote the firm innovation ability and economic growth (Simonen and Mccann, 2008; Nie L. B. et al., 2022). Through technology transfer, patent licensing, and other ways, explicit knowledge can flow to firms. Then universities can not only guide firm technological innovation but also stimulate the enthusiasm of internal R&D investment and enhance the ability of independent innovation. On the other hand, universities have a unique educational function. They can provide talent support for the industry, exchange and interact with neighboring groups through certain spatial flows, share innovative ideas and R&D intentions in the frontier of related fields, and affect the innovation performance of surrounding firms through this tacit knowledge spillover (Prencipe et al., 2020). By providing feedback on the market demand of firms in the technology transfer process, universities can guild R&D activities and talent cultivation. The interaction between production and learning helps improve regional innovation (Tseng et al., 2020).

### Heterogeneous knowledge spillover channels in universities and innovation in firms

R&D activities in universities are inconsequential sources of technology innovation. The theory of regional innovation systems suggests that these activities will perform knowledge spillover effects on other innovation subjects. Generally, there are four ways of knowledge spillover in universities: R&D collaboration, patent citations, technology transfer, and knowledge stock.

(1) R&D collaboration

In recent years, due to the increase in technological complexity and novelty in innovation activities (Barbieri et al., 2020), it is hard for firms to achieve technology breakthroughs by themselves. There is a new trend that firms utilize external resources for cooperative research (Chesbrough, 2006; Nie P-. Y. et al., 2022). Besides, firms tend to cooperate with universities and scientific research institutes instead of internal R&D activities to reduce technology transaction costs and increase innovation efficiency (Caloghirou et al., 2021). Existing studies show that industry–university–research cooperation improves firm innovation efficiency and increases firm patent application and authorization (Robin and Schubert, 2013). Further studies have found that R&D input, firm size, government subsidies, and geographical factors will affect the relationship between cooperative research and efficiency of technology innovation in industry–university–research cooperation (Szucs, 2018). Some scholars have analyzed the mechanism between industry–university–research cooperation and firm innovation performance, through joint patent technology correlation, geographical proximity, and previous relationships (Petruzzelli, 2011). However, scholars have also found that with cooperative efforts increasing, technological innovation activities become more and more dependent on collaborative innovation of industry–university–research cooperation, and the subsequent research of endogenous power is also increasingly inadequate (Sjoo and Hellstrom, 2021).

Though the dominant research focuses on the impact of industry–university–research cooperation on the firm innovation quantity, some scholars pay attention to the impact on the firm innovation quality. For example, based on the jointly-applied patents in the biotechnology field, scholars found that university–industry cooperation could promote firms to explore common technologies and strengthen basic research (Arant et al., 2019). There are three main logical ways how university–industry cooperation will improve firm innovation quality. First, university–industry cooperation relies on innovation in the basic research field, helping firms to produce more innovative and groundbreaking research (Lee et al., 2001; Belderbos et al., 2004). Second, industry–university–research cooperation can help firms share innovation costs and reduce the difficulty and risk of high-quality research (De Wit-De Vries et al., 2019). Because high-quality research requires large-scale, sustained investment in innovation and may be at higher risk, firms need to work with innovative entities such as universities to overcome low enthusiasm and low motivation to invest in innovation. Finally, university–industry collaboration enhances firm innovation ability by leveraging both explicit and implicit knowledge spillovers (Wei and Su, 2013; Wirsich et al., 2016). It is because the scientific knowledge held by universities has been often tacit knowledge, which can only be transferred through continuous exchanges in long-term cooperation.

(2) Patent citations

According to the resource dependence theory, prior knowledge relying on specific technical fields can produce higher knowledge spillover (Pfeffer, 1972; Hillman and Dalziel, 2003). Because local knowledge search and learning in specific technical tracks are conducive to the growth of the total amount of knowledge (Dosi, 1982; Cohen and Levinthal, 1990). When new ideas are combined with existing knowledge, new knowledge will be generated (Schilling and Green, 2011). Near-by patents are more likely to produce knowledge spillovers (Nemet and Johnson, 2012; Kaplan and Vakili, 2015), and far-flung technology consolidation can be costly (Todo et al., 2016) and uncertain (Kaplan and Vakili, 2015), which may inhibit the internal knowledge generation process (Gkypali et al., 2017). On the other hand, according to the technological evolution view, too much emphasis on prior knowledge leads to technological similarity and evolutionary progressivity. It may bring higher technology lock-in and path dependency in the innovation process (Burmaoglu et al., 2019). Thus, to overcome this limitation, highly differentiated but complementary knowledge in different fields is necessary for technological progress (Costantini et al., 2015; Wang et al., 2021). Important inventions tend to originate from different disciplines but inter-related (Schilling and Green, 2011). A diverse knowledge base and different technologies can have a greater influence on subsequent innovation (Battke et al., 2016). As a result, the breadth and the diversity of knowledge reserves help generate high-value research (Schoenmakers and Duysters, 2010) and lead to breakthrough innovation (Van Den Bergh, 2008).

Research on knowledge spillover mainly focuses on firm and industry levels, while there are few on universities, knowledge producers, and innovation providers (Anselin et al., 2000). In recent years, more and more attention has been paid to the role of academic research institutions in the study of knowledge spillovers, especially universities (Prencipe et al., 2020). As a basic research institution, universities put more emphasis on basic research, achieving technological breadth and external diffusion. Their research results have more technology breadth and diversity of knowledge reserves (Bong et al., 2022). Research shows that the spillover effect of R&D activities in universities is beneficial to the development of the regional economy and the construction of innovation systems in the regions where universities are located (Kang and Liu, 2021). It can be seen that universities play an important role in regional innovation through real knowledge spillovers in the form of patents and academic papers. However, there is little research on the role of microfirms, especially the influence of knowledge spillover on quantity and quality.

(3) Technology transfer

The transformation of scientific and technological achievements in universities is a kind of direct technology transfer, through which universities trade their technology to firms for commercialization directly (Scuotto et al., 2020; Bengoa et al., 2021). It is an important way to serve society as a source of science and technology (Mansfield and Lee, 1996). Many studies show that technology transfer in universities can stimulate regional innovation vitality and economic growth (Escribano et al., 2009). The impact of the transformation of scientific and technological achievements in universities on regional innovation is mainly reflected in two aspects (Nie et al., 2021). Firstly, the transformation of scientific and technological achievements in universities promotes knowledge flow and diffusion among different subjects. It is useful for the optimal combination of science and technology with other production factors, thus promoting technological innovation and economic development (Villani et al., 2017). The scientific and technological achievements of universities flow to firms through the forms of technology transfer and patent licensing. The technology transfer modes are not only simple property transactions but also include technical services and support. It can not only guide firms' technological innovation, but also stimulate their internal R&D investment enthusiasm and independent innovation capabilities. Secondly, the reverse effect of technology transfer from universities and talent demand also affects regional innovation (Gong et al., 2020). In the process of technology transfer, in addition to financial returns, universities can also obtain further R&D information through market feedback channels to guide their scientific research activities and personnel training. In addition, the reverse demand effect of technology will further promote the overall level of innovation.

However, if firms over-rely on the transformation of innovation achievements, and fail to engage in the technological R&D process, it will be difficult to raise innovation levels, or even fall into innovation inertia, and the cycle of introducing, lagging, and reintroducing (Gong et al., 2020). Some scholars have found that the transformation of results in China's public sector comes with too much administrative intervention and lacks sufficient market-based operation to play a role in the technological progress of technology recipients (Huang and Chi, 2013). Therefore, it is worth further discussing whether the transformation of scientific and technological achievements in universities can have a positive effect on regional innovation and whether there are differences in innovation quantity and quality.

(4) Knowledge stock

The impact of green technology stock in universities on firm innovation performance is a special way of tacit knowledge spillover. Tacit knowledge is not easy to transplant and transfer (Szulanski, 2000). It generally exists in the R&D activity process and minds of R&D subjects with a relatively immovable character, and it will not disappear due to the transfer of knowledge to other subjects. Under relatively closed and static conditions, knowledge subjects do not have the motivation to actively transfer knowledge. However, with the advantage of geographical proximity, firms around universities can obtain invisible knowledge that is difficult to perceive as visible to the outside world, by means of observation, communication, and learning. They can also obtain experience and lessons in the R&D process in universities, thus forming a competitive advantage (Nie et al., 2021).

Generally, the geographical proximity between regions facilitates the knowledge diffusion of universities, especially for non-coding knowledge and tacit knowledge, which also explains the reason why the abundant scientific research achievements of universities in the region positively affect firms' innovation performance (Fuentes and Dutrenit, 2016). Geographical proximity provides convenience for knowledge diffusion and interactive learning between universities and firms. It has a positive effect on innovation performance through tacit knowledge (Feldman, 1994). However, over-connection of geographical proximity leads to over-reliance on peripheral innovation resources and neglect of external knowledge acquisition. As a result, technology lock-in and disadvantages in the long-term development of firm innovation activities will be made (Singh and Marx, 2013).

Previous literature has analyzed the impact on subsequent innovation from a single form of knowledge spillover. Although it provides preliminary empirical evidence and theoretical insights for understanding technology-driven green technology innovation, there are still some improvements that can be made. First, patent citations, R&D collaboration, technology transfer, and knowledge stock all belong to the basic forms of knowledge spillover, which outlines the path for the driving effect of university innovation activities on firm innovation. However, in reality, technology drive should be a collection of multiple technologies applied. The scattered and independent studies fail to reflect the complete effect of the university innovation drive, not reflect the precise drive intensity of each spillover channel, which leads to biased results. Second, most of the existing literature focuses on the impact of the quantity of subsequent innovation, less on the impact of innovation quality. The quantity of innovation is generally conceived as reflecting the impact efficiency of knowledge spillovers, and the quality of innovation is reflecting the impact effect. Therefore, a systematic and differentiated analysis of the two is necessary. Finally, the existing literature explores the impact on firm green technology innovation from the perspectives of regulatory-push and demand-pull approaches. Few works of literature have come from the technology-driven perspective on the impact of firm green innovation, failing to examine in depth the impact mechanism and external conditions on green innovation. Above all, the green technology activities of firms are an important strategy to combat environmental pollution and achieve sustainable economic and social development, while universities are the source of technological innovation. The existing literature lacks the exploration of the relationship between the two roles and the influence mechanism. This gap provides an opportunity for the study of this article.

## Empirical design

### Data and sample

The research focus of this paper is on how the heterogeneous knowledge spillover channels of green technology innovation of universities affect the quantity and quality of subsequent green technology innovation of firms in the region. Therefore, the collection of green patents and the construction of technology connections between universities and focal firms is critical.

Firstly, according to the criteria for classifying green patents (Ardito et al., 2019; Miremadi et al., 2019) in the Green Inventory of the International Patent Classification, we manually compiled the IPC numbers mentioned in this inventory and formed a sample set of patent numbers. Using the State Intellectual Property Office and the Chinese Patent Data Service Platform database, we matched the IPC numbers of the patent sample set and download green patents filed in China from 2010 to 2019.

Secondly, the current right holder and geographical location information were obtained using Python. The patents were classified into universities, firms, research institutions, individuals, and others according to the subjects to which they belonged (Nie et al., 2021). In addition, the information was gathered from the subject and geographic information at the provincial level. Thus, on the dependent variable level, the data set of the green patent application of firms in the region was constructed. On the independent variable level, the data set of green patents belonging to universities in the region were constituted.

Thirdly, because this paper discusses the role of green innovation activities in promoting green innovation technology of firms, we need to further analyze green knowledge spillover channels. The patents of universities were selected and processed as follows: (1) The quantity of patentee was judged on whether it was a joint patent application, and then the information was extracted and separated by Python. If it was accessed to be a joint patent application between universities and firms, their names were further retrieved in Google Maps to find out whether it was a joint application activity within the same province, and then the data was aggregated at the provincial level. (2) Manual analysis of changes in the legal status of university patents was made. For the data where the transfer of patent rights and technology licensing occurred, the transferor and the receiver of the patent were extracted. Then, find out the data where the transferor was a university and the receiver was a firm, and determine whether it was a local science and technology achievement transformation behavior. (3) To analyze the flow direction of green patent citation in universities, the backward-cited patents of university green patents were separated by Python and downloaded to the patent database. After extracting the information of the right holder and geographic location, we matched them with university-focused patents to determine whether they were knowledge spillover behaviors in this region.

Finally, the data of the control variables mentioned in the paper came mainly from databases such as China Statistical Yearbook, China Statistical Yearbook of Science and Technology, and China Statistical Yearbook of Industrial Economy. To eliminate the influence of price, this paper used 2010 as the base period and deflated the relevant indicators by using the price index. In addition, because of the serious deficiency of Tibetan data, We eliminated that data. The above steps finally formed the green innovation data set of 30 Chinese provinces from 2010 to 2019.

### Variables

#### Dependent variable

##### Green innovation in firms (GreenP)

Local firms' green innovation can be measured in a variety of ways, including revenue from new product sales, patent applications, and market competitiveness. But patents remain one of the most direct products of innovation activities (Rabier, 2017). Since patent data offers such considerable benefits as comprehensiveness, generality, and accessibility, several scholars give priority to patents when analyzing a firm's innovation performance or evaluating its innovation capabilities (Guan and Liu, 2016). In line with this convention (Hall and Harhoff, 2012; Sears and Hoetker, 2014), the quantity of green innovation *(Quantity)* is measured by the number of green patents applied by firms in each province in the current year, and the quality of green innovation *(Quality)* is measured by the proportion of invention patents to the total patents in the current year.

#### Independent variables

There are four channels as our independent variables: R&D collaboration, patent citations, technology transfer, and knowledge stock.

##### R&D collaboration (joint)

A collaboration between universities and local firms is typically manifested through joint patent applications. We identify joint application patents through the information of patent applicants.

##### Patent citations (cited)

This channel refers to the use by local firms of knowledge produced by universities. We use the forward citations of a patent to measure its influence. The more a patent is cited, the greater its impact on subsequent innovation. Since patents granted in the future are unavailable and there is variability in patent disclosure and duration, it is not possible to enumerate all forward citations for our patent sample. Besides, patents granted earlier are more likely to be searched and cited, whereas the most recent ones are not. For the considerations of truncation and comparability (Nemet and Johnson, 2012), we use the number of citations within 5 years after the patent is publicized as a proxy variable for patent citations.

##### Technology transfer (TechTrans)

The technology transfer (e.g., patent commercialization, license, etc.) is the third channel. We use text comparisons to determine whether a patent has been transferred. Specifically, we utilize keywords to identify patents where the original applicant is a university and where the legal status has been marked with transfer or license. We obtain the geographical location of the patent transfer and determine whether it is local commercialization *via* string matching. Finally, we aggregate the number of local commercialization of university green patents.

##### Knowledge stock (KnowStoc)

This channel refers to the firm use of green innovation stock in local universities. Innovation is complicated, and novel discoveries are typically kept under wraps. Firms in the vicinity of universities are more likely to acquire their tacit knowledge through interactions such as personnel flow, conferences, training, and so on. As a result, the green patent stock of local universities is chosen as the fourth spillover channel.

#### Control variables

We also have controlled the influence of regional and firm-level factors on innovation activities. At the regional level, to accurately control the level of regional economic development, we use GDP (GDP) and population (Pop) as the proxy variables. Besides, when faced with fierce market competition, firms tend to have a high demand for innovative products. Intense market competition needs to acquire external knowledge. Therefore, we use a market-oriented index (Mkt) to control the level of development of the regional market. To grasp the vitality of the local technology trading market, the turnover of the provincial technology market (TechMart) has also been controlled.

At the firm level, the number of industrial firms (FirmNum) above the local scale has been controlled, and the R&D personnel (R&DPer) and R&D expenditure (R&DExp) of firms above scale is used to accurately grasp the R&D activities of firms in sub-regions. Finally, to control the influence of the time factor and the type of institution, the year of patent disclosure (Year) has been controlled and the time fixed effect model has been made.

### Empirical approach

In the analysis of the impact of heterogeneous knowledge spillover channels on firm innovation, we use panel data from 31 provinces in China for empirical analysis (excluding Hong Kong, Macao, and Taiwan). Firstly, the Hausman test of heterogeneous knowledge spillover channels on firm innovation performance shows that the fixed-effect model is more accurate. However, there is heteroscedasticity in the BP test. Because the Hausman test cannot reveal heteroscedasticity well, the LSDV method is used to solve the individual heterogeneity (Xia et al., 2020). Besides, the estimation results are consistent with the fixed effect, and the heteroscedasticity problem can be solved effectively.

## Results

### Descriptive statistics

Generally, the quantity of firm green innovations in China is small, and the quality of inventions is low (see Table1). During the observation years, the average number of firm patent applications is only 1,169, and the number of patent applications in universities is 337 in all provinces in the same period, which shows a big gap in the ratio of the firm patent to university patent in traditional innovation. Besides, the overall quality of green patent applications of firms is low, with < 36% intervention patents, and there are few exploratory innovation achievements. From the perspective of heterogeneous knowledge spillover channels, due to the restriction of geographical distance, the number of joint patent applications between universities and local firms is higher than the number of joint patent applications with distant firms. However, there is little difference in patent commercialization, which shows that the transformation of scientific and technological achievements is affected by many factors. With the development of information technology, the patent knowledge overflow breaks space limit, and the amount of patent citations in different places is three times that of local patent citations.

**Table 1:** Summary statistics.

**Variable**	**Definition**	***N***	**Mean**	**p50**	**sd**	**Min**	**Max**
**Dependent variables**							
*F*_quantity_	The quantity of green patents applied for by firms in each province over the years	300	1,169	515	1,727	5	11,346
*F*_quality_	The quality of green patents applied for by firms in each province over the years	300	0.36	0.35	0.1	0.15	0.7
**Independent variables**							
Joint	The quantity of green patents jointly applied by universities and local firms in each province over the years	300	12.35	5	18.28	0	146
Cited	The quantity of green patents in universities cited by local firms in each province over the years	300	108.8	39	183.6	0	1,186
TechTrans	The quantity of green patents in universities commercialized locally by province over the years	300	0.97	0	2.02	0	17
KnowStoc	Stock of green patents in universities by province over the years	300	306.5	180	359.2	0	2,623
**Control variables**							
GDP	GDP of each province	300	22,860	17,314	19,141	1,144	10,7987
Population	Population of each province	300	4,549	3,834	2,725	563	11,521
FirmNum	Number of firms in each province	300	426,997	289,833	444,708	13,225	303,7617
Mkt	A market-oriented index to control the level of development of regional market	300	6.46	6.36	1.88	2.33	10
TechMart	Provincial technical market transaction volume (100 million yuan)	300	337.4	95.18	702.3	0.57	5,695
R&DPer	R&D personnel of firms above provincial scale	300	84,074	46,369	114,385	1,157	642,490
R&DExp	Research and development expenditure of firms above provincial scale (million)	300	31,848	17,074	42,002	401.9	231,486
Province	Provinces of universities and firms	300	38.37	39	16.47	11	65
Year	The year when a patent was publicized.	300	2015	2015	2.88	2010	2019

Finally, to solve the extreme value influence, some variables are logarithmized. So all variables are in a relatively concentrated range without the interference of extreme outliers. In our group regression, the test of variance inflation factor (VIF) for all variables is < 10, which indicates that the effect of multicollinearity on the results could be excluded.

### The heterogeneous impact of knowledge spillover channels

In this section, we examine the impact of heterogeneous knowledge spillover channels of universities on the green technology innovation of local firms. The results are shown in Table 2. After including control variables and fixed time and region effects, models 1–5 demonstrate the effects of different spillover channels on the quantity of subsequent innovation. Overall, patent citations, technology transfer, and knowledge stock positively contribute to the quantity of subsequent innovation. Models 6–10 show the impact of different spillover channels on the quality of subsequent innovation. In general, R&D collaboration and knowledge stock have a significant incentive effect on the quality improvement of subsequent innovation.

**Table 2:** The heterogeneous impact of knowledge spillover channels in universities on the quantity and the quality of green innovation.

**D.V**.	**Innovation quantity**					**Innovation quality**				
	**(1)**	**(2)**	**(3)**	**(4)**	**(5)**	**(6)**	**(7)**	**(8)**	**(9)**	**(10)**
Joint	−0.002				−0.042	0.028***				0.030***
	(0.052)				(0.054)	(0.006)				(0.007)
Cited		0.083**			0.061		0.001			−0.019**
		(0.039)			(0.045)		(0.009)			(0.008)
TechTrans			0.143***		0.098**			0.015		0.013*
			(0.034)		(0.041)			(0.010)		(0.007)
KnowStoc				0.141***	0.104**				0.024***	0.034***
				(0.037)	(0.042)				(0.008)	(0.012)
Control variables	Y	Y	Y	Y	Y	Y	Y	Y	Y	Y
Year FE	Y	Y	Y	Y	Y	Y	Y	Y	Y	Y
Province FE	Y	Y	Y	Y	Y	Y	Y	Y	Y	Y
Constant	−3.728***	−4.121***	−3.532***	−2.698***	−3.622***	0.837***	0.633***	0.668***	0.832***	1.006***
	(0.620)	(0.454)	(0.406)	(0.630)	(0.500)	(0.078)	(0.092)	(0.085)	(0.075)	(0.059)
*N*	300	300	300	300	300	300	300	300	300	300
*R* ^2^	0.899	0.901	0.900	0.902	0.906	0.179	0.148	0.143	0.162	0.251

Standard errors in parentheses.

*p < 0.1, **p < 0.05, ***p < 0.01.

In model 1, the coefficient of R&D collaboration is positive but insignificant (β = −*0.002, p* > *0.1*). In model 6, the coefficient of R&D collaboration is positive and significant (β = *0.028, p*<*0.01*). This indicates that R&D collaboration between universities and firms in the green technology field cannot promote the quantity of green technology innovation of firms, but it can promote their innovation quality. Firms and universities unconsciously disseminate and share technical knowledge when jointly exploring technologies (Subramanian et al., 2013). It can enrich firms' scientific knowledge base. However, it can also lead to over-reliance on external innovation resources and reduce the motivation and initiative of innovation within the firm. This is negative for the advancement of firm innovation quantity. The joint development of innovative products by multiple entities can raise the technical requirements for innovative products, thus inhibiting the increase in the quantity of overall innovation. On the other hand, R&D collaboration can significantly stimulate innovation quality. This is because technology exchange and cooperation help firms better assess the quality and value of innovation. Rich scientific and technological knowledge also facilitates firms to better evaluate the quality and value of innovations and filter ideas with insufficient technical content and economic benefits. This facilitates leading firms to invest resources into valuable inventions, enabling them to develop the most advanced technologies that are difficult for other firms to imitate (Belderbos et al., 2016), thus improving the quality of innovation.

In model 2, the coefficient of patent citations is positive and significant (β = *0.083, p*<*0.05*). In model 7, the coefficient of R&D collaboration is positive but insignificant (β = *0.001, p* > *0.1*). This indicates that the use of green technologies of universities in the innovation process by local firms can contribute to the total quantity of innovation, but does not promote the quality. In the process of firm innovation, the reference of specific knowledge depends on the innovation activities of prior knowledge spillover in specific technology fields. Resource dependence theory suggests that local knowledge search and local technological learning in a specific technological track facilitate the growth of total knowledge (Dosi, 1982; Cohen and Levinthal, 1990). New knowledge is generated when new ideas are combined with existing knowledge (Schilling and Green, 2011). In particular, patents with more recent technological proximity are more likely to generate innovative ideas. However, the technological evolutionary perspective contends that too much emphasis on prior knowledge leads to technological similarity and evolutionary progressivity. In turn, it will lead to higher technological lock-in and path dependency (Burmaoglu et al., 2019). In addition, more distant technology integration may lead to high costs (Todo et al., 2016), and uncertainty (Kaplan and Vakili, 2015), and thus negatively affect innovation quality promotion.

In model 3, the coefficient of technology transfer is positive and significant (β = *0.143, p*<*0.01*). In model 8, the coefficient of technology transfer is positive but insignificant (β = *0.015 p* > *0.1*). This suggests that the green technology in the local science and technology achievements transformation behavior can greatly promote the innovation quantity in the region, but insufficient incentive for innovation quality. This is because when a firm purchase external technology, it can obtain the property right information and technical data of the patent at one time. On this basis, firms develop derivative technologies in multiple directions around the core technology, expand in different technology scenarios, and further enhance the amount of subsequent innovation. It can also stimulate the enthusiasm for internal R&D investment and innovation. However, if there's too much dependence on external technologies and a lack of deep participation in the technology development process, it will restrict the internal research promotion. It may even lead to innovation inertia and fall into the cycle of introduction, lagging, and reintroduction, making it difficult to improve the subsequent innovation quality.

In model 4, the coefficient of knowledge stock is positive and significant (β = *0.141, p*<*0.01*). In model 9, the coefficient of knowledge stock is positive and significant (β = *0.024, p*<*0.01*). This indicates that the green technology stock of universities has a significant impact on the quantity and quality promotion of green innovation in the region. The innovation performance of firms is not only affected by their innovation ability but also driven by external forces. The technological innovation of universities can lead to knowledge spillover, which will affect third-party R&D activities. In the regional environment, the individual R&D cost of firms is reduced. The innovation achievements of universities in the region flow into firms to promote the direct use of innovation results through explicit knowledge spillover. In addition, it will also interact and exchange with the surrounding groups through the flow of tacit knowledge and talents in the spatial scope. This will accelerate the dissemination of successful experiences and failed lessons in the R&D process, thus promoting the technological innovation activities of firms. Therefore, the green technology of universities in the region can provide an a priori knowledge base for the quantitive improvement of firm innovation, and also promote the quality improvement of innovation through the exchange of tacit R&D experiences.

Considering that the impact of university knowledge spillover on subsequent innovation has a certain lag, the dependent variable is lagged by one period in Table 3 to verify the time factor. Models 1-5 show the empirical results of the innovation quantity lagged by one period. The results show that the impact coefficients and significance of different channels are consistent with the empirical results in Table 2. However, the impact coefficients are significantly larger, suggesting that there is a time-lagged amplification effect. Models 6–10 show the empirical results of innovation quality with a one-period lag. The patent citation channel has a significant negative effect on innovation quality. This indicates that technological R&D activities that transition to the use of existing innovations do not improve the quality of innovation and R&D, and this drawback shows a lag. Technology transfer has a lag on research quality improvement, suggesting that firms need time for technology deconstruction and redevelopment after acquiring technology property rights through market transactions. The results show that joint application and knowledge stock coefficients are not significant. It indicates that there is no time lag in the research results of universities on the firm innovation quality promotion.

**Table 3:** The heterogeneous impact of knowledge spillover channels in universities on the quantity and quality of green innovation (t+1).

**D.V**.	**Innovation quantity (t**+**1)**					**Innovation quality (t**+**1)**				
	**(1)**	**(2)**	**(3)**	**(4)**	**(5)**	**(6)**	**(7)**	**(8)**	**(9)**	**(10)**
Joint	0.017				−0.007	0.010				0.009
	(0.035)				(0.034)	(0.008)				(0.008)
Cited		0.144***			0.118***		−0.016**			−0.022**
		(0.039)			(0.044)		(0.008)			(0.009)
TechTrans			0.082*		0.055			0.015**		0.017**
			(0.047)		(0.045)			(0.008)		(0.008)
KnowStoc				0.201***	0.115*				0.006	0.018
				(0.062)	(0.069)				(0.015)	(0.018)
Control variables	Y	Y	Y	Y	Y	Y	Y	Y	Y	Y
Year FE	Y	Y	Y	Y	Y	Y	Y	Y	Y	Y
Province FE	Y	Y	Y	Y	Y	Y	Y	Y	Y	Y
Constant	−4.994***	−5.147***	−5.176***	−3.361**	−4.312***	0.127	0.075	0.164	0.293	−4.994***
	(1.600)	(1.575)	(1.539)	(1.710)	(1.653)	(0.228)	(0.205)	(0.260)	(0.301)	(1.600)
*N*	270	270	270	270	270	270	270	270	270	270
*R* ^2^	0.707	0.730	0.708	0.721	0.736	0.196	0.215	0.206	0.191	0.233

Standard errors in parentheses.

*p < 0.1, **p < 0.05, ***p < 0.01.

### Additional analysis

#### The moderating effect of firm absorptive capacity

The absorptive capacity theory holds that firms need to have a knowledge base that matches the new knowledge to identify, absorb, and utilize new knowledge (Cohen and Levinthal, 1990). As the recipient of knowledge spillovers from universities, the absorptive capacity of firms is the premise and guarantee of searching, digesting, and integrating external knowledge, better digestion, and use of external resources to enhance competitive advantage (Engelman et al., 2017). In this section, the per-capita years of education are used as the proxy of absorptive capacity to further study the impact of different levels of absorptive capacity levels on firm heterogeneous knowledge spillover channels in universities.

The empirical results in Table 4, models 1–4, show that higher absorptive capacity can significantly enhance the impact of university–industry cooperation and patent citation on firm innovation quantity. When controlling for other spillover channels simultaneously in model 5, this incentive effect still exists, but it crowds out the promotion effect of technology transfer on subsequent innovation. In terms of innovation quality in models 6–10, high absorptive capacity significantly improves the effect of university knowledge spillover on the quality of subsequent innovation, but the effect on other spillover channels is not significant. This effect remains consistent after controlling for other channels in model 10. Overall, the absorptive capacity of firms has obvious differences in the moderating effect of different knowledge spillover channels, suggesting that we should precisely design the corresponding forms of university–industry cooperation for firms with different absorptive capacities.

**Table 4:** The moderating effect of firm absorptive capacity.

	**Innovation quantity**					s**Innovation quality**				
	**(1)**	**(2)**	**(3)**	**(4)**	**(5)**	**(6)**	**(7)**	**(8)**	**(9)**	**(10)**
**Independent variables**										
Joint	−0.488**				−1.602***	0.086*				0.266***
	(0.222)				(0.246)	(0.048)				(0.080)
Cited		−0.227			−0.622***		−0.037*			−0.037
		(0.217)			(0.239)		(0.022)			(0.029)
TechTrans			0.432		1.436***			−0.023		−0.003
			(0.300)		(0.375)			(0.061)		(0.082)
KnowStoc				−0.171	1.091***				−0.042	−0.172
				(0.254)	(0.221)				(0.055)	(0.109)
**Moderator variable**										
AC	−0.207***	−0.245**	−0.041	−0.392**	−0.197	0.029*	−0.008	0.020**	−0.031	−0.054
	(0.066)	(0.098)	(0.026)	(0.167)	(0.151)	(0.017)	(0.018)	(0.010)	(0.051)	(0.071)
**Interactions**										
Joint*AC	0.059**				0.178***	−0.007				−0.028*
	(0.026)				(0.030)	(0.005)				(0.009)
Cited*AC		0.037*			0.072***		0.006**			0.004***
		(0.022)			(0.023)		(0.003)			(0.003)
TechTrans*AC			−0.030		−0.140*			0.005		0.002
			(0.037)		(0.044)			(0.007)		(0.009)
PatStoc*AC				0.046	−0.101				0.008	0.021
				(0.029)	(0.028)				(0.008)	(0.014)
Control	Y	Y	Y	Y	Y	Y	Y	Y	Y	Y
Year FE	Y	Y	Y	Y	Y	Y	Y	Y	Y	Y
Province FE	Y	Y	Y	Y	Y	Y	Y	Y	Y	Y
Constant	−2.629*	−1.519	−3.855***	0.676	−0.496	−0.496	0.560**	0.935***	0.616***	1.195**
	(1.497)	(1.369)	(1.254)	(2.029)	(1.704)	(1.704)	(0.225)	(0.282)	(0.177)	(0.596)
*N*	300	300	300	300	300	300	300	300	300	300
*R* ^2^	0.895	0.898	0.897	0.899	0.907	0.907	0.194	0.174	0.171	0.172

Standard errors in parentheses.

*p < 0.1, **p < 0.05, ***p < 0.01.

#### The moderating effect of environmental regulation level

Environmental regulation and firm green innovation performance have been the focus of academic attention (Porter and Vanderlinde, 1995; Jaffe and Palmer, 1997), and the research perspective focuses on whether environmental regulation promotes or inhibits firm green innovation, and what policy design tools are better at raising environmental standards. However, the effect of environmental regulation on the firm between universities and firms in the field of green innovation and on the innovation performance of firms has not been fully demonstrated and empirically tested. Therefore, this section further explores the relationship between heterogeneous knowledge spillovers and firm innovation performance through the environmental regulation level in different regions. At present, there are two kinds of measuring methods for environmental regulation: single index measuring method and comprehensive index measuring method. Since the single index measuring method has a bias problem (Becker, 2005), in this paper, reference has been used to select industrial wastewater emissions, sulfur dioxide emissions, and smoke and dust emissions to build a comprehensive index to measure the environmental regulation.

Table 5 validates the moderating effect of environmental regulation on different knowledge spillover channels, models 1–5 validate the impact on innovation quantity, and models 6–10 validate the impact on innovation quality. The results show that the higher level of environmental regulation has a positive effect on the promotion of innovation quantity in universities, and the four knowledge spillover channels, while the environmental regulation level has no significant influence on the innovation activities of universities and firms. This suggests that when under strict environmental regulation, firms need to search widely and absorb external knowledge edge to improve innovation ability as quickly as possible (Chesbrough, 2003). They also need to improve technological innovation achievements and deal with environmental problems through the digestion and absorption of external knowledge (Pakura, 2020). Therefore, a high level of environmental regulation plays a positive regulating role in the four knowledge spillover channels. However, under the pressure from the outside world, firms absorb universities' knowledge spillover results, most of which is incremental innovation based on the original innovation, thus it has a limited promotion effect compared with exploratory innovation. As a result, the quality of innovation has not improved significantly. In models 5 and 10, we consider the interaction of spillover channels. Under high environmental regulations, the contribution of technology transfer to innovation quantity is insignificant with a negative direction, but joint applications can improve the quality of subsequent innovations.

**Table 5:** The moderating effect of environmental regulation level.

	**Innovation quantity**					**Innovation quality**				
	**(1)**	**(2)**	**(3)**	**(4)**	**(5)**	**(6)**	**(7)**	**(8)**	**(9)**	**(10)**
**Independent variables**										
Joint	−0.035				0.030	0.025***				0.009
	(0.060)				(0.072)	(0.009)				(0.012)
Cited		−0.016			−0.078		0.016***			−0.006
		(0.043)			(0.049)		(0.006)			(0.015)
TechTrans			0.061*		0.151***			0.011		0.010
			(0.033)		(0.054)			(0.008)		(0.014)
KnowStoc				0.086***	0.124**				0.028***	0.042**
				(0.027)	(0.049)				(0.007)	(0.019)
**Moderator variable**										
ER	−0.054	−0.068*	−0.002	−0.110***	−0.143***	0.002	0.005	0.004	0.007	0.027
	(0.035)	(0.037)	(0.018)	(0.037)	(0.045)	(0.009)	(0.009)	(0.004)	(0.017)	(0.025)
**Interactions**										
Joint *ER	0.028***				0.049**	0.001				0.013*
	(0.009)				(0.034)	(0.003)				(0.007)
Cited*ER		0.019***			0.017**		−0.000			0.005
		(0.005)			(0.016)		(0.002)			(0.007)
TechTrans *ER			0.027*		−0.010			0.002		−0.002
			(0.014)		(0.021)			(0.003)		(0.005)
KnowStoc *ER				0.024***	0.041*				−0.000	−0.014
				(0.005)	(0.024)				(0.003)	(0.012)
Control	Y	Y	Y	Y	Y	Y	Y	Y	Y	Y
Year FE	Y	Y	Y	Y	Y	Y	Y	Y	Y	Y
Province FE	Y	Y	Y	Y	Y	Y	Y	Y	Y	Y
Constant	−4.256***	−4.231***	−4.430***	−3.411***	−3.321***	0.734***	0.617***	0.589***	0.806***	0.826***
	(0.431)	(0.422)	(0.443)	(0.400)	(0.528)	(0.090)	(0.098)	(0.109)	(0.089)	(0.077)
*N*	240	240	240	240	240	240	240	240	240	240
*R* ^2^	0.892	0.893	0.892	0.894	0.899	0.182	0.162	0.148	0.167	0.222

Standard errors in parentheses.

*p < 0.1, **p < 0.05, ***p < 0.01.

#### The heterogeneity analysis of regional factors

Because of China's vast territory, there are regional differences in the level of economic and social development and technological innovation ability among regions, which may lead to regional heterogeneity in the impact of spillover channels of universities on firm innovation. The purpose of this section is to grasp the impact of knowledge spillover channels on local firm innovation and to improve the universality. The economic development level of each province in China is divided into east, middle, and west. Models 1–3 in Table 6 analyzes the impact of heterogeneous knowledge spillover channels on the firm innovation quantity in the three regions, and models 4–6 analyzes the impact on the innovation quality.

**Table 6:** The heterogeneity analysis of regional factors.

	**Innovation quantity**			**Innovation quality**		
	**(1)**	**(2)**	**(3)**	**(4)**	**(5)**	**(6)**
	**East**	**Middle**	**West**	**East**	**Middle**	**West**
Joint	−0.012	−0.062**	−0.084*	0.023	0.032*	0.049***
	(0.040)	(0.026)	(0.082)	(0.017)	(0.019)	(0.019)
Cited	0.074	0.046	0.040	−0.011	−0.042***	−0.003
	(0.052)	(0.039)	(0.076)	(0.011)	(0.005)	(0.014)
TechTrans	−0.043	−0.055	0.296***	0.018***	0.014	0.041
	(0.040)	(0.102)	(0.099)	(0.006)	(0.024)	(0.026)
KnowStoc	0.347***	0.127	0.365***	−0.017	0.071**	−0.008
	(0.118)	(0.115)	(0.112)	(0.036)	(0.036)	(0.023)
Control variables	Y	Y	Y	Y	Y	Y
Year	Y	Y	Y	Y	Y	Y
Constant	−1.218	−13.465***	2.727	0.533***	1.561***	0.357
	(1.448)	(1.066)	(1.859)	(0.129)	(0.441)	(0.423)
*N*	110	80	110	110	80	110
*R* ^2^	0.962	0.935	0.796	0.529	0.475	0.163

Standard errors in parentheses.

*p < 0.1, **p < 0.05, ***p < 0.01.

Our findings are as follows. First, R&D collaboration has a significant effect in the mid-western region, but not in the eastern region. It has a negative crowding-out effect on the innovation quantity in the central and western regions and a positive incentive effect on the innovation quality. This is because the innovation level of firms in mid-western regions is generally weak, and the opportunity to cooperate with universities can make full use of innovation resources in universities and significantly improve their innovation quality level. At the same time, the technology level difference often depends on the university's innovation cooperation, and on the contrary, the innovation quality has not obtained remarkable promotion.

Second, the adoption of university patents by industry has no significant impact on the development of regional technology innovation, and even harms the innovation quality in the central region. Patents citation, one of the channels through which university research results spill out to the outside world, breaks the geographical distance limit and gains knowledge spill-out in a wider scope with the development of information technology. There is no clear impact on the region, and too much knowledge in universities is cited by local firms, which is an inappropriate time and will lead firms into path dependence and hinder their breakthrough innovation development.

Third, commercialization of scientific and technological achievements locally can enhance firm innovation quantity in the western region and the innovation quality in the eastern region. Commercialization means that firms need to pay more for the exclusive ownership of the technology. Firms in the western region tend to value the research results because of their limited conditions. Based on this technology, more innovative outputs would be obtained through in-depth experimental development. Compared with the high level of firm innovation ability in the eastern region, it has a stronger absorptive capacity. Therefore, it can improve innovation quality by fully absorbing the bottom technology.

Lastly, the knowledge stock of regional universities plays a significant role in promoting the firm innovation quantity and quality. Due to the convenience brought by geographical proximity, firms can acquire the knowledge of innovation imperceptibly through the exchange of personnel and the learning of experience and lessons.

On the whole, there are obvious regional differences in the impact of heterogeneous knowledge spillovers in universities on firm innovation. The conclusions of this section can provide a reference for the formulation of precise innovation support policies and the selection of cooperation ways between firms and universities.

## Discussion

Green technologies have been considered important to address environmental pollution and achieve high-quality economic and social development, which is highly valued and expected by the state and society. However, the development of green technology is very slow due to its high investment and low revenues. Existing studies focus on fields such as regulatory-push and demand-pull approaches (Horbach et al., 2012), but there is a gap in the role of technology push (Koch and Simmler, 2020). Therefore, based on the innovation diffusion theory and grounded on the realistic pursuit of high-quality economic and social development in China, this paper constructs a multi-dimensional path model of knowledge spillover in universities. Moreover, it also explores whether university innovation activities can promote green technology in firms and how to. It is among the first to investigate the impact of heterogeneous knowledge spillover channels on green innovation activities in local firms and make an in-depth analysis of differential effects of knowledge spillover channels on the firm innovation quantity and quality. In doing so, it extends previous research on enriching innovation diffusion theory, analyzing the mechanism of knowledge spillover channel based on different forms of university–industry cooperation, and constructing a multidimensional path model of knowledge spillover in universities.

These results help to advance the way researchers think about the role of firm innovation and, more specifically, about heterogeneous knowledge spillover channels. This paper indicates that the local commercialization of scientific and technological results in universities and the knowledge stock improve the innovation quantity and quality of local firms (Fuentes and Dutrenit, 2016). Heterogeneous knowledge spillover channels in universities have a differentiated impact on the increase of local firms' innovation quantity. On the one hand, R&D collaboration cannot boost innovation quantity but inhibits it to some degree. Firms that cite public patents also cannot boost innovation quantity. On the other hand, the commercialization and knowledge stock of the scientific and technological achievements in the region play a significant role in promoting the total amount of firm innovation. This is contrary to the prevailing research findings on university–industry collaboration. Existing research suggests that once a company has engaged in R&D collaboration with a university research institute, it will inevitably have a positive impact on its innovation output. However, we find that only the technology transfer and the knowledge stock can promote the firm innovation quantity. Therefore, to stimulate the development of innovation activities, the corresponding choice of university–industry cooperation should be emphasized.

Our results further suggest that in firms that collaborate with a (local) public institution, knowledge stock can stimulate the improvement of the firm subsequent innovation quality (Robin and Schubert, 2013). However, firms that cite public patents have an inhibitory effect on the improvement of firm innovation quality. Heterogeneous knowledge spillover channels in universities have a differentiated impact on the increase of innovation quality in local firms. On the one hand, our research finds that the joint application of industry and university compensates for the quality of subsequent innovation, though it inhibits the increase of the overall innovation quantity of firms. Moreover, the knowledge stock of local universities not only promotes firm innovation quantity but also innovation quality. On the other hand, technology spillovers from universities in the form of patents cited by firms can provide intellectual support to firms but are not conducive to improving innovation quality. Thus, improvement in innovation quality is more complex and comprehensive than previous literature would suggest. There are obvious differences in the quantity and quality of firm innovation through heterogeneous knowledge spillover channels in universities, which provides an important reference for supporting policy-making and selecting university–industry cooperation forms.

To characterize the impact of heterogeneous knowledge spillover channels on firm green technology innovation, we further investigate the absorptive capacity of knowledge acquisition, the environmental regulation level in the particular context of green innovation, and the regional heterogeneity of different economic development levels. It is found that the high absorptive capacity of firms improves the effect of university-industry collaboration and patent citations on the innovation quantity. High absorptive capacity can moderate the effect of patent citation on the improvement of innovation quality. Besides, the more strict environmental regulation, the more significant the incentive effect of knowledge spillover on the increase of green innovation quantity of firms. However, there is no such effect on innovation quality improvement. Moreover, there are obvious regional differences in the impact of the knowledge spillover channel on firm innovation in different regions of economic development level. This paper provides further evidence for the existing research on the impact of knowledge spillovers theory and firm innovation, discusses the effective ways to stimulate university knowledge spillover and firm innovation activities, and explains how to stimulate firm technological innovation and development through public R&D.

### Theoretical implications

Our work contributes to the literature on local knowledge spillovers of public R&D in several ways. Firstly, we contribute to the literature that empirically studies the impact of heterogeneous knowledge spillover channels on subsequent innovation, enriching and refining the innovation diffusion theory. It is generally believed that public R&D can promote the development of innovation activities of peripheral firms through knowledge spillovers. However, the research on how universities affect firms is often ignored. Based on Chinese green patent data, this paper discusses the effects of heterogeneous knowledge spillovers on the quantity and quality of innovation activities in the surrounding firms. The conclusion expands the depth of innovation diffusion theory and provides solutions for university–industry cooperation.

Secondly, our work contributes to the literature that distinguishes different knowledge spillover channels from the perspective of explicit and implicit technology flow. It also expands the research breadth and deepens knowledge spillover theory. Existing knowledge spillovers are often based on spatial economics to analyze the impact of inter-regional knowledge flow on innovation, and a few pay attention to different types of knowledge spillovers. This paper studies different types of public R&D knowledge spillover channels and makes a detailed analysis of the spillover effects of each channel. By integrating the technology flow perspective, this study expands the breadth and depth of research on knowledge spillover pathways. It also helps to provide empirical support for stimulating public R&D spillovers to promote peripheral innovation.

Our third contribution to the literature is that we comprehensively measure the impact of knowledge spillovers on subsequent innovation from the dimension of quantity and quality. We bring people's attention to both innovation performance and market value and extend the boundary of knowledge spillovers theory. Dialectically, quantity and quality of innovation are two aspects of a problem. Quantity reflects innovation efficiency, while quality reflects innovation effect. Previous studies have focused more on the quantitative dimension, less on the quality dimension, and less on both. Therefore, they cannot fully reflect the impact of knowledge spillovers on subsequent innovation (Cai and Li-Ping, 2017). This study provides a comprehensive empirical analysis of quantity and quality, which not only accurately reflects the impact on subsequent innovation, but also better guides innovation practice and improves the overall spillover effect of university innovation.

Our last contribution to the literature is that we provide strong evidence that public R&D can promote both the motivation of surrounding firms to innovate and their level of research, and the use of knowledge flows in collaboration. The study furthers and enriches the theoretical foundation of “industry-university-institute” cooperation. Previous research has focused on the drivers of public R&D, which have a positive impact on subsequent innovation. Universities with high R&D intensity tend to be more willing to collaborate with firms on research. They also tend to promote their development in the form of talent cultivation and facilitation of talent mobility. On this basis, this study focuses on patents that represent applied research results to examine the impact of public R&D applied technologies on the quantity and quality of technological innovation in firms, to more directly transform it into real productivity.

### Implications for practitioners

(1) From the perspective of knowledge sources, the knowledge spillover of university R&D activities plays a comprehensive role in promoting firm innovation activities, both innovation quantity and quality. However, there are significant differences in the impact of different spillover channels on firm innovation. Therefore, the role of public R&D as the source of technological innovation needed to be strengthened by the state. Governments at all levels should increase the investment in innovation resources to accelerate knowledge spillover and transformation. They should also improve the innovation evaluation criteria and the incentive mechanism for the transformation. Besides, the incentive effect of public R&D on the spillover of surrounding firms should be introduced into the assessment mechanism. Secondly, the government should deepen the reform of the science and technology system, and provide precise support for university–industry cooperation. The policy focus should be shifted from the industry–university–research cooperation which only focuses on the single explicit knowledge flow, to equal emphasis on explicit and tacit knowledge flows.

(2) From the perspective of knowledge recipients, improving firms' innovation ability and building a good learning mechanism are conducive to knowledge recipients' active learning ability. On the one hand, the absorptive capacity of firms has a significant impact on the spillover effects of different channels, which indicates firms' innovation capacity is the core of technological innovation performance. Firms should adopt a more open, diversified, and pioneering form, strengthen their innovation ability, and break the dependence on the inherent innovation process and innovation mode. On the other hand, the innovation output of firms is closely related to the R&D activities of universities. Different forms of knowledge acquisition channels have influenced innovation output. Combined with its development status, technological innovation ability, and other factors, firms should explore an industry cooperation mode with convenient communication, smooth information, and flexible methods. They should also seek to establish an open and smooth channel of knowledge and talent flow with universities and scientific research institutes, to achieve synergy and complementarity between internal R&D forces and external R&D resources. As a result, a double breakthrough in innovation quantity and innovation quality is likely to be achieved.

(3) From the perspective of the knowledge spillover environment, we should establish close and diversified university–industry cooperation networks, and develop university–industry technology platforms and technology intermediary services for knowledge diffusion. Besides, the government also needs to facilitate the smooth and convenient flow of public R&D results to the industry, create an internal and external environment suitable for knowledge spillover and reduce the search cost, communication cost, and potential risks of firm knowledge acquisition. At the same time, the limitation of the current innovation policy should be optimized. Cooperation between industry and university is not only a mode of interaction but also a lot of implicit spillover forms. Thirdly, the evaluation standard of “value quantity, ignore quality” should be avoided in policies. Attention needs to be paid to the input of different knowledge spillovers to the difference between quantity and quality, optimizing the innovation market mechanism and legal environment.

### Limitations and future research

Inevitably, this paper still has some deficiencies and improvements. First, though this paper uses typical patent data of knowledge spillovers in universities, there are also other forms of scientific and technological achievements of universities, such as academic papers and talent cultivation. Future studies can include relevant data to test the accuracy and generalisability of the results. Second, this research considers the impact of university knowledge spillover channels on firm innovation but neglects the influence of the depth and breadth of university–industry cooperation on the quantity and quality of innovation. Further, we do not consider the acquisition cost and knowledge acquisition paths of firm knowledge in heterogeneous spillover channels. Future research can optimize the university–industry cooperation mode, adjust internal and external factors, and combine the breadth and depth of cooperation to achieve the balance of different spillover channels. Third, macro-provincial data does not consider the mechanism of individual firms' impact on the knowledge acquisition channels of universities at the microlevel. Besides, we have not looked at the role played by micro factors such as different R&D intensity, development stage, nature of firms, etc. Therefore, to enhance the practicality of research conclusions, future research can be targeted at the specific factors of micro-firms. Fourth, there are interactions among the four spillover channels, so it is necessary to know how the interactions affect innovation outcomes. Although the impact of their interaction on innovation quantity and quality is examined in the paper, the micro-mechanisms and dynamic processes of their interaction and impact on innovation outcomes need to be distinguished and verified with mathematical models more precisely.

## Conclusion

Based on the theory of innovation diffusion and the perspective of industry–university–research cooperation, grounded on the reality of the pursuit of high-quality economic and social development in China, this paper constructs a multidimensional path model of knowledge spillover in universities. We discuss the impact of heterogeneous knowledge spillover channels on the green innovation activities of local firms and analyze the differential impact of different knowledge spillover channels on the quantity and quality of innovation. The conclusion provides a new solution and growth path for the weak green technology development in China and a more accurate reference basis for the choice of university–industry cooperation patterns. It is found that there is a great difference in the quantity and quality of the subsequent innovation, which can be summarized as follows. First, the R&D collaboration channel does not promote the quantity of subsequent innovation but promotes the quality. Second, on the contrary, the commercialization of university patents channel can increase the quantity of innovation but have no significant effect on the quality. Third, the effect of the spillover channel cited by the firms is similar to that of patent commercialization, but there is a big difference between the two coefficients. Fourth, the non-specific channels of knowledge stock in universities play a significant role in the improvement of the quantity and quality of the subsequent industrial innovation.

We further find that under the special circumstances of green technology innovation, environmental regulation can enhance the impact of the four spillover channels on the quantity of subsequent innovation, but does not improve the quality. Moreover, the moderating effect of the absorptive capacity on the spillover channel has been examined. With high absorptive capacity, the patent citation spillover channel plays a more important role in improving the quantity and the quality of innovation. At the same time, R&D collaboration channel with high absorptive capacity region can stimulate the firm innovation quantity.

## Data availability statement

The original contributions presented in the study are included in the article/supplementary material, further inquiries can be directed to the corresponding author/s.

## Author contributions

LBN: writing—original draft preparation, visualization, investigation, and data curation. HG: conceptualization, methodology, and supervision. DXZ: validation and writing—reviewing and editing. XPL: conceptualization, software, and writing—reviewing and editing. MYC: software and editing. All authors read and approved the final manuscript.

## Funding

The work was financially supported by the National Social Science Fund of China (21BGL266).

## Conflict of interest

This authors declare that the research was conducted in the absence of any commercial or financial relationships that could be construed as a potential conflict of interest.

## Publisher's note

All claims expressed in this article are solely those of the authors and do not necessarily represent those of their affiliated organizations, or those of the publisher, the editors and the reviewers. Any product that may be evaluated in this article, or claim that may be made by its manufacturer, is not guaranteed or endorsed by the publisher.
